# Polyimide-Based PolyHIPEs Prepared via Pickering High Internal Phase Emulsions

**DOI:** 10.3390/polym11091499

**Published:** 2019-09-13

**Authors:** In-Ho Song, Dong-Min Kim, Ju-Young Choi, Seung-Won Jin, Kyeong-Nam Nam, Hyeong-Joo Park, Chan-Moon Chung

**Affiliations:** Department of Chemistry, Yonsei University, Wonju, Gangwon-do 26493, Korea; segunda@nate.com (I.-H.S.); dmkimr@yonsei.ac.kr (D.-M.K.); cjy0510@yonsei.ac.kr (J.-Y.C.); jinsw0906@yonsei.ac.kr (S.-W.J.); nkn001@yonsei.ac.kr (K.-N.N.); hyeongjoo1016@yonsei.ac.kr (H.-J.P.)

**Keywords:** porous polyimide, polyHIPE, Pickering high internal phase emulsion

## Abstract

Pyromellitic dianhydride (PMDA) and 4,4′-oxydianiline (ODA) oligoimide particles and PMDA-ODA poly(amic acid) salt (PAAS) were synthesized and used as stabilizers to prepare oil-in-water Pickering high internal phase emulsions (HIPEs). The stability of the Pickering HIPEs was investigated by dispersion stability analysis. Polyimide-based polyHIPEs could be prepared through freeze-drying and subsequent thermal imidization of the Pickering HIPEs. The characteristics of the polyHIPEs, including their morphology, porosity, thermal decomposition temperature, and compression modulus, were investigated. The thermal decomposition temperature (*T*_10_) of the polyHIPEs was very high (>530 °C), and their porosity was as high as 92%. The polyimide-based polyHIPEs have the potential to be used in high-temperature environments.

## 1. Introduction

Porous materials can be used for numerous applications, such as gas storage and separation membranes, catalysts, and insulating materials, due to their characteristics, including their low density and wide surface area [1,2,3,4,5]. Porous polymers can be prepared by diverse synthetic routes, are easily processed, and can be designed to have a variety of chemical functionalities or pore structures [6]. Among the various methods of preparing porous polymers, the use of high internal phase emulsion (HIPE) as a template is a convenient method of producing a polymer having high porosity and permeability. HIPEs are emulsions that contain more than 74 vol. % internal phase based on the total emulsion volume. PolyHIPEs are emulsion-templated porous polymers synthesized within the HIPEs [6,7,8,9]. The typical preparation method of polyHIPEs includes radical polymerization of a monomer which is dissolved in an external and/or internal phase in a HIPE [6,7,8,9,10,11,12,13,14,15]. PolyHIPEs have a unique open cell structure with two kinds of macropores: droplet-templated macropores called voids and their interconnecting macropores called windows [6,7,8,9]. The unique macropore structure of polyHIPEs with high permeability has been evaluated for applications such as adsorbent or separation membranes of gasses and ions [16,17], catalysts [18,19], tissue engineering [20] and heat/sound insulation [21].

To enhance the applicability of the polyHIPEs in high-temperature environments, a variety of approaches has been proposed. PolyHIPEs based on poly(aryl ether sulfone) [22], a crosslinked polymer from 2-nitroresorcinol/cyanuric chloride [23] and a fluoropolymer [24], were reported to have improved thermal stability. Some organic–inorganic nanocomposites also have been developed as thermally stable polyHIPEs [25,26,27,28]. Those heat-resistant polyHIPEs were reported to be stable up to 240 to 400 °C. However, it is desirable to develop polyHIPEs having higher thermal stability for wider applications in high-temperature industry. 

A Pickering emulsion is an emulsion prepared using solid particles as a stabilizer. Pickering emulsions have good stability because solid particles strongly adsorb on the oil–water interface [29,30]. Compared to conventional HIPE stabilized with a molecular surfactant, a Pickering HIPE has advantages in that it can be stabilized with a relatively small amount of particles and the use of solid particles may add additional physical properties to the polyHIPE [6,7,8]. In contrast, to prepare a HIPE using a conventional molecular surfactant, a large amount of surfactant of up to 30% of the external phase is often required, and removal of the surfactants from the polyHIPE may require additional costs [7]. 

In this work, we have developed a polyimide-based polyHIPE possessing high thermal stability. Polyimide is known to have excellent thermal stability, mechanical properties, and chemical resistance [31,32]. Here, polyHIPEs were prepared via Pickering HIPEs stabilized with oligoimide particles and poly(amic acid) salt (PAAS). The Pickering HIPEs were subjected to lyophilization and thermal imidization to produce polyimide-based polyHIPEs. The characteristics of the polyHIPEs, including their morphology, thermal properties, mechanical properties, and porosity, were investigated.

## 2. Materials and Methods 

### 2.1. Materials 

Pyromellitic dianhydride (PMDA) and 4,4′-oxydianiline (ODA) were purchased from Sigma-Aldrich (St. Louis, MO, USA). 1-Methyl-2-pyrrolidone (NMP), pyridine, acetic anhydride, triethylamine (TEA), and cyclohexane were purchased from DUKSAN (Ansan, Gyeonggi-do, Korea). Acetone was purchased from SK Chemicals (Seongnam, Korea). An aluminum mold with a cubic hole (20 mm × 20 mm × 20 mm) was made and used to freeze-dry the Pickering HIPEs.

### 2.2. Instruments 

Oligoimide particle dispersions were prepared in water using an ultrasonicator (VCX750, Sonic & Materials, Newtown, CT, USA) at 20 kHz and an amplitude of 20%. HIPEs were prepared using a homogenizer (T18, IKA, Staufen, Germany). Freeze-drying of HIPEs was conducted using a lyophilizer (FD 2.5, Heto Lab Equipment, Allerød, Denmark).

### 2.3. Characterization

Infrared spectra were obtained using a Fourier transform infrared (FT-IR) spectrophotometer (Spectrum One B, Perkin Elmer, Waltham, MA, USA). The optical microscopy images of HIPE droplets were obtained using a microscope (BX-51, Olympus, Tokyo, Japan). The sizes of the emulsion droplets were analyzed using a CCD camera (HK6U3Cool, KOPTIC, Seoul, Korea) equipped in the microscope and image analysis software (HKBasic, KOPTIC). The average diameter was determined from a data set of at least 1000 measurements for each sample. The inherent viscosities were determined using a viscometer (Cannon-Fenske type, Sigma-Aldrich, St. Louis, MO, USA) at a concentration of 0.5 g/dL in concentrated sulfuric acid at 30 °C. Dispersion stability analysis (Turbiscan Lab Expert, Formulaction, France) was conducted with monochromatic light at *λ* = 880 nm and 25 °C for 24 h to determine the backscattering of the Pickering emulsion. The morphology of the polyHIPEs was observed by a field emission scanning electron microscope (FE-SEM) (SU-70, Hitachi, Ltd., Tokyo, Japan) at an accelerating voltage of 30 KV. The porosity of the polyHIPEs was analyzed using a mercury porosimeter (AutoPore IV 9520, Micromeritics Instrument Co., Norcross GA, USA). Thermal analyses were carried out under a N_2_ atmosphere using thermogravimetric analysis (TGA) (Discovery TGA 55, TA instrument, Inc., New Castle, DE, USA) at a heating rate of 10 °C/min. The compression modulus measurement of the polyHIPEs was performed using a universal testing machine (UTM) (QC-505M1, Daeha Trading Co., Ltd., Seoul, Korea) with a loading of 1 mm/min.

### 2.4. Preparation of PMDA-ODA Oligoimide Particles

PMDA-ODA oligoimide particles were prepared according to a previously reported procedure (Scheme S1) [33]. PMDA (10.9 g, 50 mmol) and water (200 mL) were added to a 500 mL two-neck round-bottom flask fitted with a condenser, and the flask was heated in an oil bath at 100 °C. ODA (10.0 g, 50 mmol), pyridine (20.3 mL, 250 mmol), and acetic anhydride (9.4 mL, 100 mmol) were added to the flask, and the resulting mixture was stirred at 100 °C under N_2_ gas for 24 h. Then, the reaction mixture was poured into distilled water. The product was collected by filtration, washed with acetone, and dried under vacuum. FT-IR (KBr, cm^−1^) PMDA-ODA: 1777 (imide C=O asymmetric stretch), 1725 (imide C=O symmetric stretch), 1382 (imide C–N stretch). Inherent viscosity: 0.06 dL/g.

### 2.5. Preparation of PMDA-ODA Poly(amic acid) Salt (PAAS)

The synthesis of PAAS is illustrated in Scheme S2. ODA (10.0 g, 50 mmol) and NMP (200 mL) were added to a 500 mL two-neck round-bottom flask fitted with a mechanical stirrer. After the ODA dissolved, PMDA (10.9 g, 50 mmol) was added, and the resultant mixture was stirred for 6 h at room temperature to produce poly(amic acid) (PAA). After TEA (10.1 g, 100 mmol) was added, the resulting solution was stirred for 6 h and then poured into acetone for reprecipitation. The product was collected by filtration and dried under vacuum. FT-IR (KBr, cm^−1^) PMDA-ODA PAAS salt: 1671 (amide C=O stretch), and 1550 (carboxylate C=O and amide N–H stretch). Inherent viscosity of PAA (in NMP): 0.53 dL/g.

### 2.6. Preparation of Pickering HIPEs and PolyHIPEs

An illustration of the preparation procedure is presented in Figure S1 and Scheme S3. PMDA-ODA particles (0, 1, 3, 5, or 10 wt.% with respect to PAAS/TEA solution) were added to a PAAS (0, 2, 4, 6, or 8 wt.% with respect to TEA solution)/TEA solution (water:TEA = 98:2 by vol.). The dispersion was subjected to ultrasonication for 30 min. HIPE was prepared by adding cyclohexane as an internal phase (75, 80, or 85 vol.% with respect to the total mixture) in an aqueous dispersion and subsequent homogenization. The homogenization was conducted at 20,000 rpm after a homogenizer tip was slightly immersed in the dispersion. The homogenization was performed until cyclohexane did not remain visible and usually was finished within 3 min. The resulting Pickering HIPE was filled into the mold and frozen in liquid nitrogen. The frozen samples were placed into a freeze-dryer to remove the ice crystal template at −50 °C. Thermal imidization of the freeze-dried HIPEs was conducted at 100, 200, 300, and 400 °C. The samples were kept at each temperature for 1 h.

## 3. Results and Discussion

### 3.1. Synthesis of PMDA-ODA Oligoimide Particles and PAAS

The PMDA-ODA particles were synthesized in water in the presence of pyridine and acetic anhydride (Scheme S1). The viscosity of the particles was measured to be relatively low, so they are considered to be an oligomer. The pristine PMDA-ODA particles prepared in water had a plate-like shape and a size range of 1 to 5 μm (Figures S2 and S3). Ultrasonication of the particle dispersion in water for 10 or 30 min broke the particles into much smaller 0.7 to 1.8 or 0.1 to 0.5 μm sized particles, respectively. In this study, the 0.1 to 0.5 μm sized particles were only used to prepare HIPEs because they afforded the most stable HIPEs, compared to the other sized particles. The PMDA-ODA poly(amic acid) salt (PAAS) was synthesized via the reaction of PMDA and ODA in NMP, treatment with TEA, and subsequent reprecipitation in acetone (Scheme S2).

**Figure 1:**
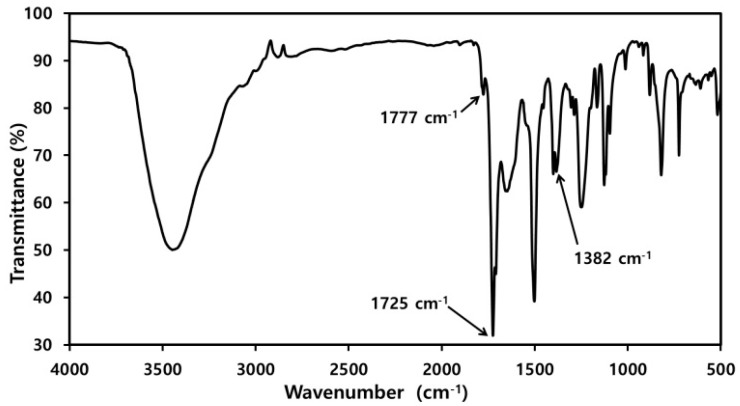
FT-IR spectrum of pyromellitic dianhydride-4,4′-oxydianiline (PMDA-ODA) oligoimide.

**Figure 2:**
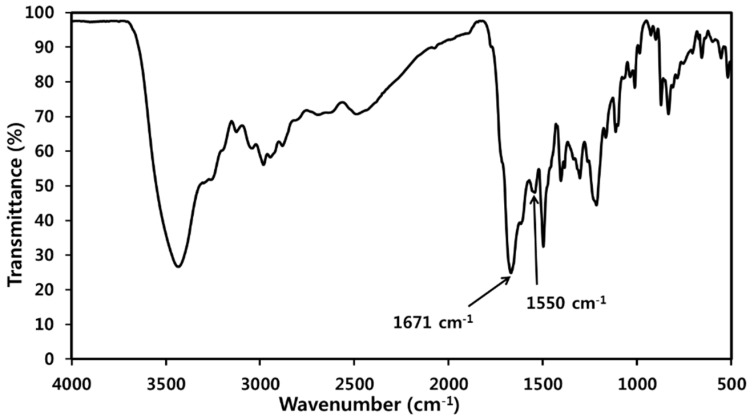
FT-IR spectrum of PMDA-ODA poly(amic acid) salt (PAAS).

FT-IR analysis was used to confirm the structure of the synthesized PMDA-ODA particles (Figure 1) and PAAS (Figure 2). The structure of the oligoimide was identified by the absorption bands due to asymmetric imide C=O stretching at 1777 cm^−1^, symmetric imide C=O stretching at 1725 cm^−1^, and imide C–N stretching at 1382 cm^−1^. On the other hand, the PAAS showed absorption bands due to amide C=O stretching at 1671 cm^−1^ and overlapped carboxylate C–O and amide N–H stretching at 1550 cm^−1^ [34,35].

### 3.2. Preparation of Pickering HIPEs

**Figure 3:**
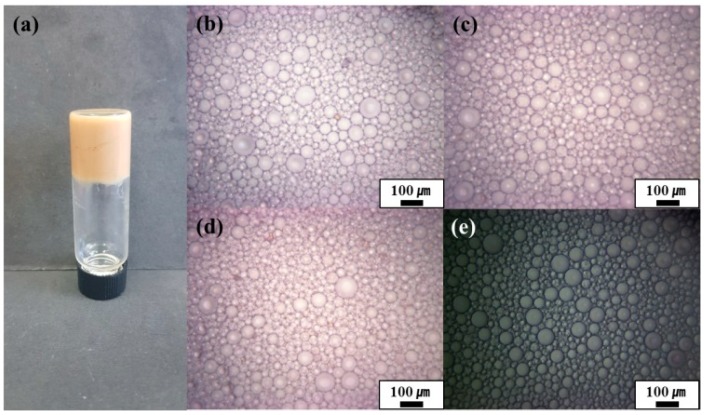
Optical micrographs of Pickering HIPEs prepared with 6 wt.% PAAS and 80 vol.% internal phase using oligoimide particle concentrations of (**b**) 1 wt.%, (**c**) 3 wt.%, (**d**) 5 wt.%, and (**e**) 0 wt.%. (**a**) A photograph of the Pickering HIPE shown in (**d**).

We previously reported that Pickering emulsions could be prepared using oligoimide particles as a stabilizer [33]. In this study, Pickering HIPEs were prepared using cyclohexane as an oil phase, and oligoimide particles and PAAS as stabilizers (Figure 3). Cyclohexane was added to the oligoimide particle/PAAS mixtures in water, and then homogenization was conducted. The particle weight percent, PAAS weight percent, and internal phase volume percent of the HIPEs were varied. Solely by using oligoimide particles, internal phase concentration increased up to 50 vol.%, but a HIPE could not be prepared. When using only PAAS, a HIPE could be prepared, but the HIPE-templated macropore structure collapsed during lyophilization (this is discussed in more detail below). Therefore, Pickering HIPEs containing PAAS as a co-stabilizer were prepared to produce the polyHIPEs [7].

**Figure 4:**
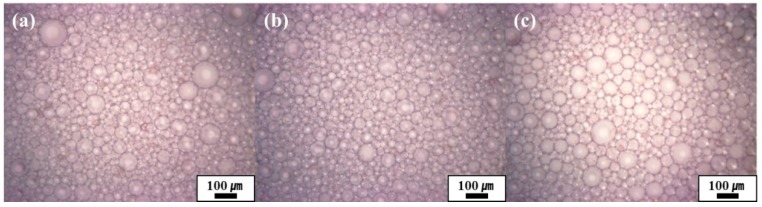
Optical micrographs of Pickering HIPEs prepared with 6 wt.% PAAS and 5 wt.% oligoimide particle concentrations with (**a**) 75 vol.% (**b**) 80 vol.%, and (**c**) 85 vol.% internal phase.

The prepared HIPEs were studied by optical microscopy (Figure 3b–e). The average sizes of the emulsion droplets according to the concentration of the oligoimide particles (0, 1, 3, and 5 wt.%) were 30 (±13), 48 (±12), 53 (±9), and 46 (±9) μm, respectively (Figure 3b–e). The average sizes of the emulsion droplets according to the concentration of the PAAS (2, 4, 6, and 8 wt.%) were 54 (±9), 49 (±8), 46 (±9), and 47 (±9) μm, respectively (Figure S4). Clear trends, according to the particle concentration or PAAS concentration, were not observed. On the other hand, the average droplet sizes of the emulsions having internal phase volume of 75, 80, and 85 vol.% were measured to be 44 (±9), 46 (±9), and 52 (±13) μm, respectively (Figure 4). 

**Figure 5:**
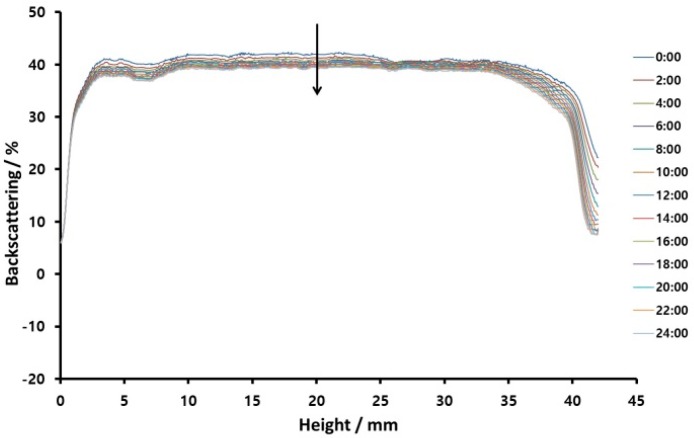
Change of backscattering of the Pickering high internal phase emulsions (HIPEs) over 24 h measured 48 h after HIPE preparation (particle 5 wt.%, PAAS 6 wt.%, internal phase 80 vol.%).

The stability of the Pickering HIPEs was investigated by measuring the backscattering intensity according to the elapsed time. A decrease of the backscattering intensity was observed over 24 h after the Pickering HIPE preparation (Figure S5). During the first 24 h, some coalescence was expected to occur based on the backscattering intensity decrease (phase separation was not observed). However, the droplet size was not significantly changed when the HIPE was observed by optical microscopy. When the measurement was performed after a stabilization time of 48 h (Figure 5), it was confirmed that there was no significant change (around 3%) of the backscattering intensity over 24 h. Therefore, the preparation of the polyHIPEs was conducted after stabilizing the Pickering HIPEs for 48 h for better reproducibility.

### 3.3. Preparation of PolyHIPEs

**Figure 6:**
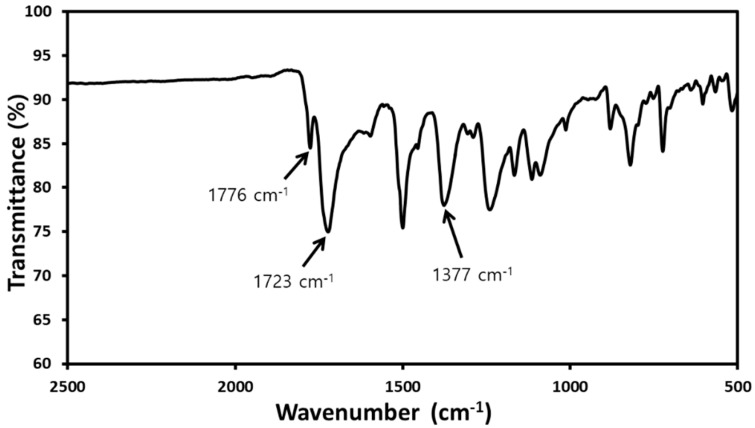
FT-IR spectrum of the prepared polyHIPE.

PolyHIPEs were prepared by freeze-drying and subsequent thermal imidization of the Pickering HIPEs (Figure S1 and Scheme S3). FT-IR analysis was used to confirm the imidization of PAAS in the HIPEs by the thermal imidization process (Figure 6). The absorption bands at 1671 and 1550 cm^−1^ in the spectrum of PAAS (Figure 2) disappeared in the imidized sample spectrum (Figure 6). The imidization of PAAS can also be confirmed by asymmetric imide C=O stretching at 1776 cm^−1^, symmetric imide C=O stretching at 1723 cm^−1^, and imide C–N stretching at 1377 cm^−1^.

### 3.4. Morphology of PolyHIPEs

**Figure 7:**
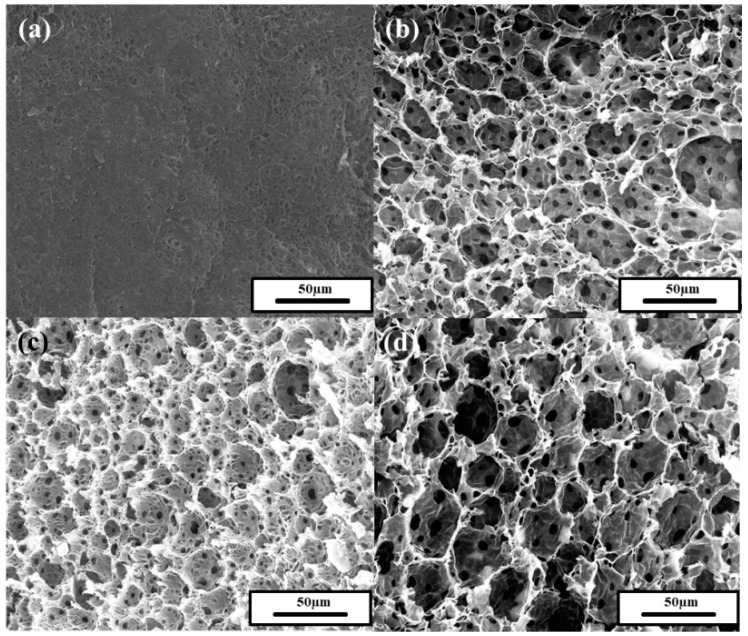
SEM images of polyHIPEs prepared with 6 wt.% PAAS concentration and 80 vol.% internal phase using oligoimide particle concentrations of (**a**) 0 wt.%, (**b**) 1 wt.%, (**c**) 3 wt.%, and (**d**) 5 wt.%.

The morphology of the prepared polyHIPEs was studied by SEM (Figure 7). Droplet-templated macropores called voids were not formed in the HIPE prepared solely using PAAS without oligimide particles (Figure 7a). The molecular surfactant (PAAS) could not sustain the droplet-templated pore structure during lyophilization and thermal imidization. In cases of the polyHIPEs prepared with both oligoimide particles and PAAS, voids and interconnecting macropores called windows were formed (Figure 7b–d). It is considered that the particles and PAAS play roles as “bricks” and “binder”, respectively. Both constituents are required to form a stable droplet-templated pore structure. 

The pore structure of the polyHIPEs is similar to those of previously reported polyHIPEs [6,7,8]. The void size was somewhat smaller than the Pickering HIPE droplet diameter. The voids were generated from the emulsion droplet, but volume contraction occurred during lyophilization and subsequent thermal imidization (Figure S6). In the thermal imidization process, PAAS liberates water and TEA (Scheme S3), resulting in volume contraction. It is postulated that the windows were formed when the internal cyclohexane left the droplets during lyophilization or when volume contraction occurred [6,9].

**Figure 8:**
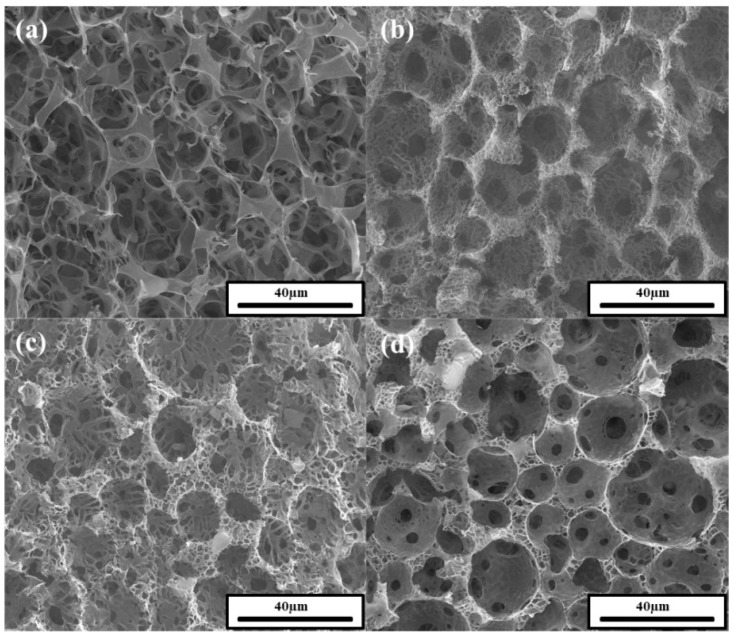
SEM images of polyHIPEs prepared at a 5 wt.% oligoimide particle concentration and 80 vol.% internal phase with PAAS concentrations of (**a**) 2 wt.%, (**b**) 4 wt.%, (**c**) 6 wt.%, and (**d**) 8 wt.%.

**Figure 9:**
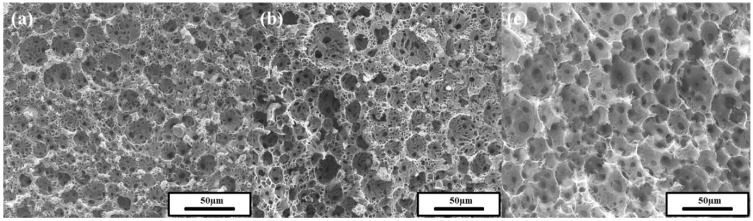
SEM images of polyHIPEs prepared with a 5 wt.% oligoimide particle concentration and 6 wt.% PAAS concentration with internal phase values of (**a**) 75 vol.%, (**b**) 80 vol.%, and (**c**) 85 vol.%.

Figure 8 shows SEM images of polyHIPEs prepared at a 5 wt.% particle concentration and 80 vol.% internal phase with different PAAS concentrations. At a 2 wt.% PAAS concentration (Figure 8a), it was difficult to determine the shape of the voids in the SEM image. Macropore voids were observed in the polyHIPEs prepared at PAAS concentrations of 4, 6, or 8 wt.% (Figure 8b–d). The void size was somewhat smaller than the Pickering HIPE droplet diameter, which is probably due to volume contraction during lyophilization and thermal imidization, as mentioned above. The size of the window did not seem to significantly change as the PAAS concentration increased. Figure 9 shows SEM images of polyHIPEs prepared at a 5 wt.% particle concentration and 6 wt.% PAAS concentration with different internal phase volume percent values.

### 3.5. Properties of PolyHIPEs

Table 1 shows the porosity, thermal decomposition temperature, and compression modulus of the polyHIPEs. The porosity was measured to be up to 92% (Sample 1). The porosity decreased as the particle, or PAAS concentration increased (Samples 1–7). This is because the solid phase occupies more volume in the polyHIPEs as the particle or PAAS concentration increases. In contrast, the porosity increased as the internal phase volume percent increased (Samples 3, 8, and 9) because the internal phase position is converted to empty space in the polyHIPEs. The preparation reproducibility was evaluated by repeating the preparation of Samples 1 to 4. The porosity of replicated Sample 1 was measured to be 91%, and the porosity trend according to particle concentration was the same as the above result, implying good reproducibility of the polyHIPE preparation. The pore size distributions were also determined by porosimetry and had a size range of 100 nm to 90 μm. A typical example is shown in Figure S7. The large pores around 90 μm in size might be derived from some large droplets shown in Figure 4.

It should be noted that thermal decomposition temperatures (*T*_10_) of the polyHIPEs were higher than 530 °C. The thermal decomposition temperatures of the polyimide-based polyHIPEs are much higher than those of conventional polyHIPEs based on other polymers, such as polystyrene, polyacrylates, and polymethacrylates [6,7,8,9,22,23,24,25,26,27,28]. Due to the high thermal stability of the polyHIPEs, they could find applications as separation membranes, insulating materials, and reaction supports for catalysts that are used in high-temperature environments.

The compression modulus of the polyHIPEs was measured using a slope of up to 20% strain in the stress–strain curves [36]. Because oligoimide particles and PAAS are constituents of the polyHIPE structure, the compression modulus increases as the oligoimide particle or PAAS concentration increases. The polyHIPEs had relatively low compression moduli because they were prepared without polymerization process of HIPE, in contrast with conventional polyHIPEs prepared by polymerization. 

## 4. Conclusions

Oil-in-water Pickering HIPEs were prepared using PMDA-ODA oligoimide particles synthesized in water and PAAS synthesized in NMP. The Pickering HIPEs had an internal phase volume ratio up to 85 vol.%. PolyHIPEs were prepared by lyophilization and thermal imidization of the Pickering HIPEs. The preparation of the polyHIPEs showed good reproducibility. The pore structure of the polyHIPEs had macropore voids and windows, which is a unique structure of a typical polyHIPE. The porosity of the polyHIPEs was measured to be up to 92% and can be controlled by varying the particle weight percent, PAAS weight percent, or internal phase volume percent. The prepared polyHIPEs had a much higher thermal stability (*T*_10_ > 530 °C) due to the imide structure of the polyimide, compared to the conventional polyHIPEs that were reported to be stable up to 240 to 400 °C. The polyimide-based polyHIPE has the potential to be used as separation membranes, insulating materials, and reaction supports for catalysts that are used in high-temperature environments. 

## Figures and Tables

**Table 1:** Properties of the polyHIPEs.

Sample No.	Particle (wt.%)	PAAS (wt.%)	Internal Phase (vol.%)	Porosity (%)	*T*_10_ (°C) ^a^	Compression Modulus (kPa)
1	1	6	80	92	555	1.14
2	3	6	80	81	564	1.46
3	5	6	80	79	543	1.91
4	10	6	80	70	540	2.50
5	5	2	80	89	530	0.14
6	5	4	80	82	535	0.61
7	5	8	80	77	552	4.38
8	5	6	75	71	557	2.86
9	5	6	85	90	533	0.47

^a^ The temperature at which a sample exhibits 10 wt.% decomposition in a nitrogen atmosphere.

## Supplementary Materials

The following are available online at https://www.mdpi.com/2073-4360/11/9/1499/s1: Scheme S1: Synthesis of PMDA-ODA oligoimide particles, Scheme S2: Synthesis of PMDA-ODA PAAS, Scheme S3: Thermal imidization of PAAS, Figure S1: Schematic illustrations of the synthesis of polyHIPE, Figure S2: FE-SEM images of PMDA-ODA particles after ultrasonication for (a) 0 min, (b) 10 min and (c) 30 min, Figure S3: PMDA-ODA particle size distribution before and after ultrasonication. The distribution curves were obtained based on the FE-SEM data (100 measurements for each sample). For each particle, the longest length was taken, Figure S4: Optical micrographs of Pickering HIPEs prepared at a 5 wt.% oligoimide particle concentration and 80 vol.% internal phase using PAAS concentrations of (a) 2 wt.%, (b) 4 wt.%, (c) 6 wt.%, and (d) 8 wt.%, Figure S5: Backscattering change of the Pickering HIPE during 24 h after HIPE preparation (particle 5 wt.%, PAAS 6 wt.%, internal phase 80 vol.%), Figure S6: Photographs of products prepared by (a) freezing of HIPE, (b) lyophilization of HIPE, and (c) thermal imidization after lyophilization, Figure S7: Pore size distribution of a polyHIPE sample prepared from a Pickering HIPE (5 wt.% oligoimide particle concentration, 6 wt.% PAAS concentration, and 80 vol.% internal phase).
