# Supplementary material for: Polyimide-Based PolyHIPEs Prepared via Pickering High Internal Phase Emulsions
Source: Polymers (Basel). 2019 Sep 13;11(9):1499. doi: 10.3390/polym11091499 (PMC6780585; doi:10.3390/polym11091499)
Supplement: Supplementary file 1 [file polymers-11-01499-s001.pdf]

Supplementary Material

Polyimide-based polyHIPEs prepared via Pickering High Internal Phase Emulsions

In-Ho Song, Dong-Min Kim, Ju-Young Choi, Seung-Won Jin, Kyeong-Nam Nam, Hyeong-Joo Park and Chan-Moon Chung *

Department of Chemistry, Yonsei University, Wonju, Gangwon-do 26493, Republic of Korea;

* Correspondence: cmchung@yonsei.ac.kr; Tel.: +82-033-760-2266

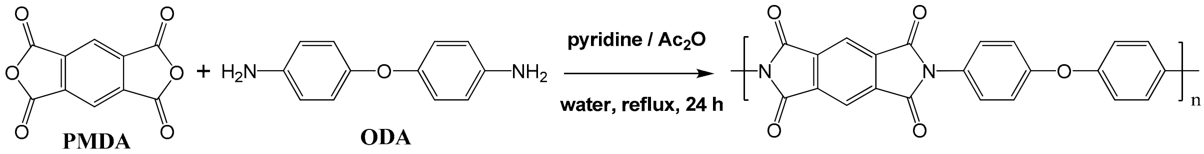

Scheme S1. Synthesis of PMDA-ODA oligoimide particles.

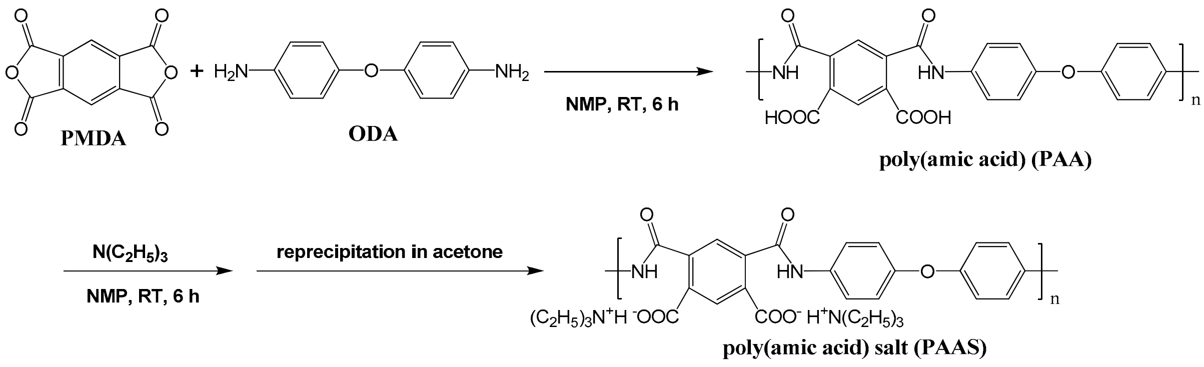

Scheme S2. Synthesis of PMDA-ODA PAAS.

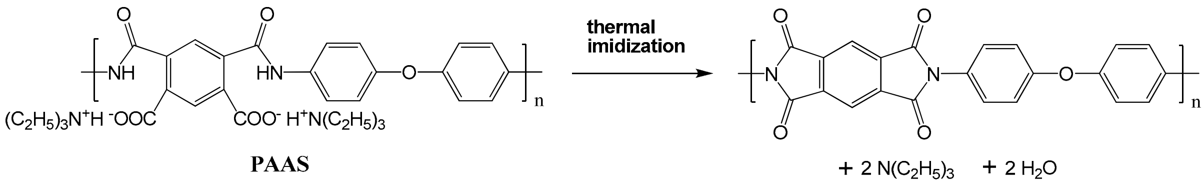

Scheme S3. Thermal imidization of PAAS.

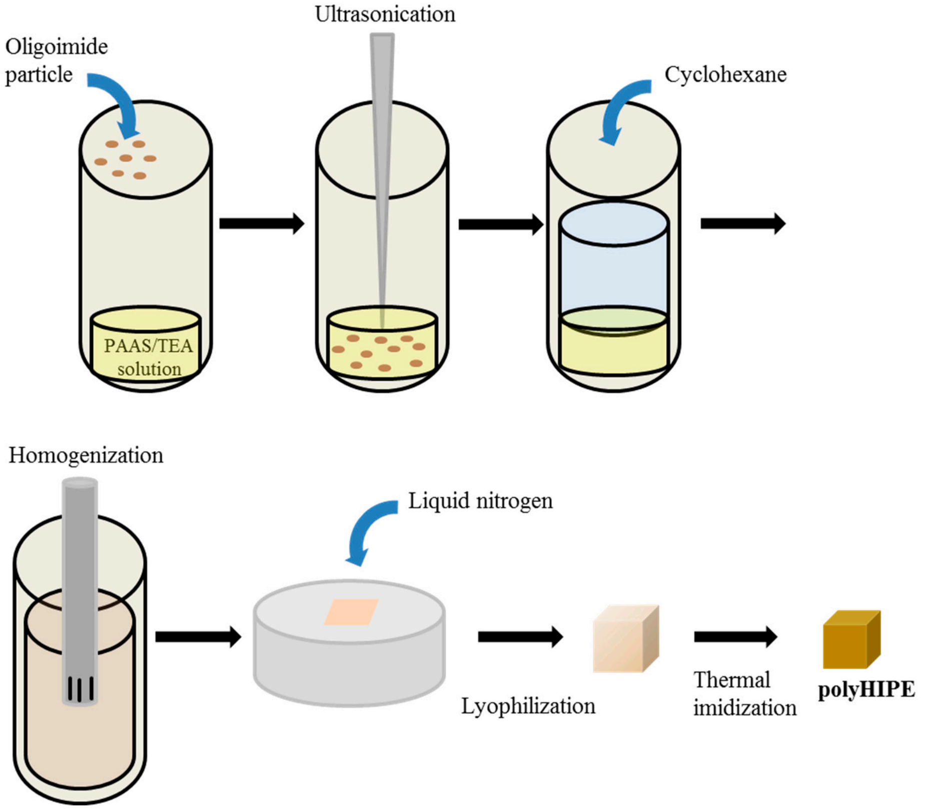

Figure S1. Schematic illustrations of the synthesis of polyHIPE.

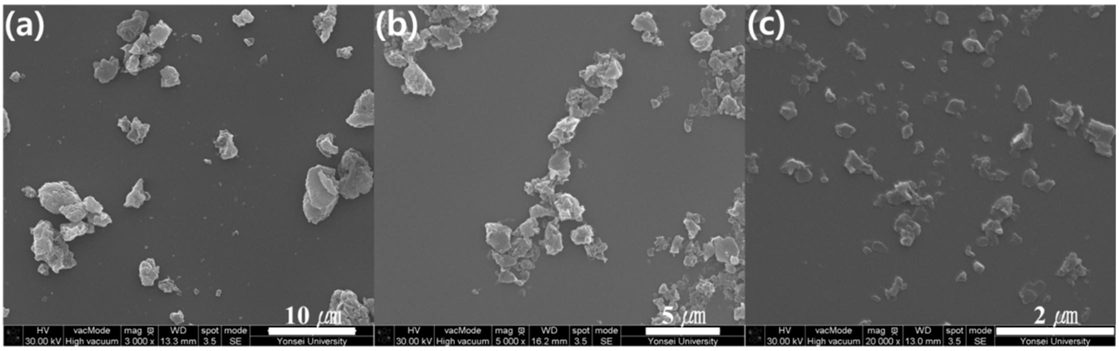

Figure S2. FE-SEM images of PMDA-ODA particles after ultrasonication for (a) 0 min, (b) 10 min and (c) 30 min.

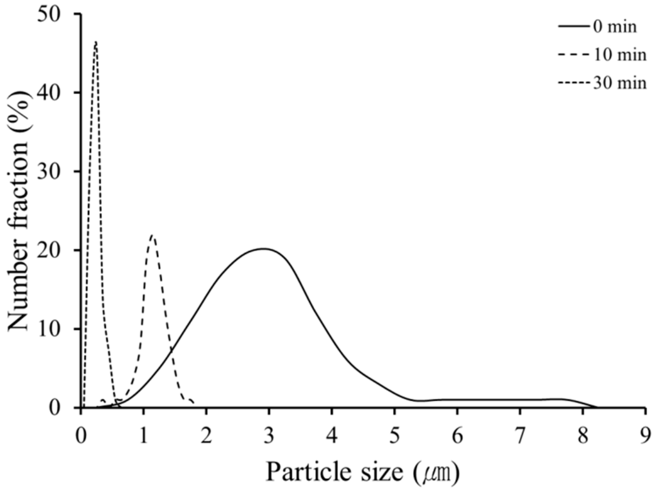

Figure S3. PMDA-ODA particle size distribution before and after ultrasonication. The distribution curves were obtained based on the FE-SEM data (100 measurements for each sample). For each particle, the longest length was taken.

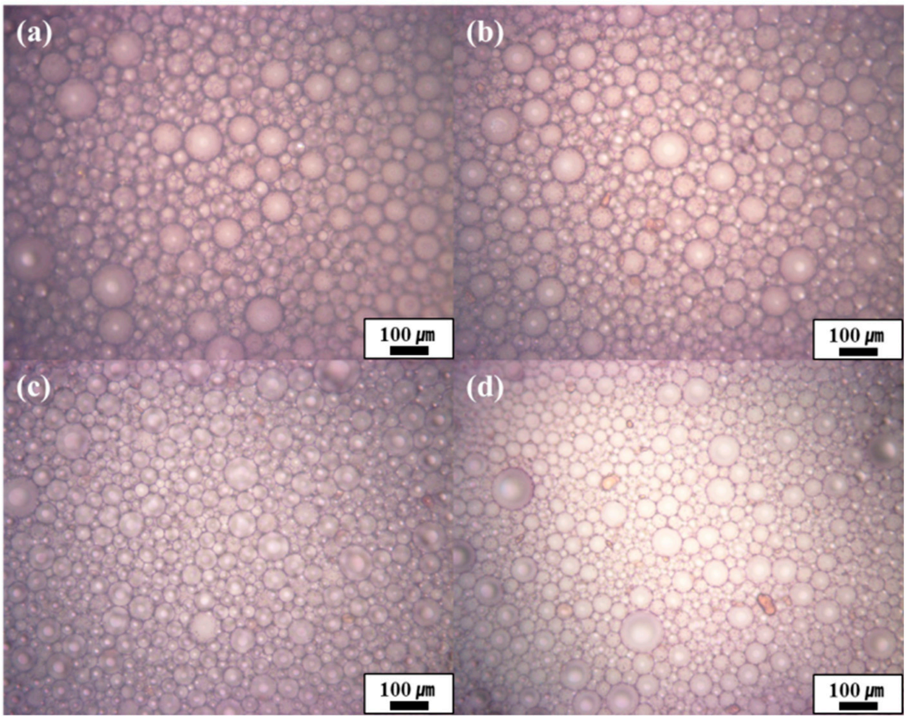

Figure S4. Optical micrographs of Pickering HIPEs prepared at 5 wt% oligoimide particle concentration and 80 vol% internal phase using PAAS concentrations of (a) 2 wt%, (b) 4 wt%, (c) 6 wt% and (d) 8 wt%.

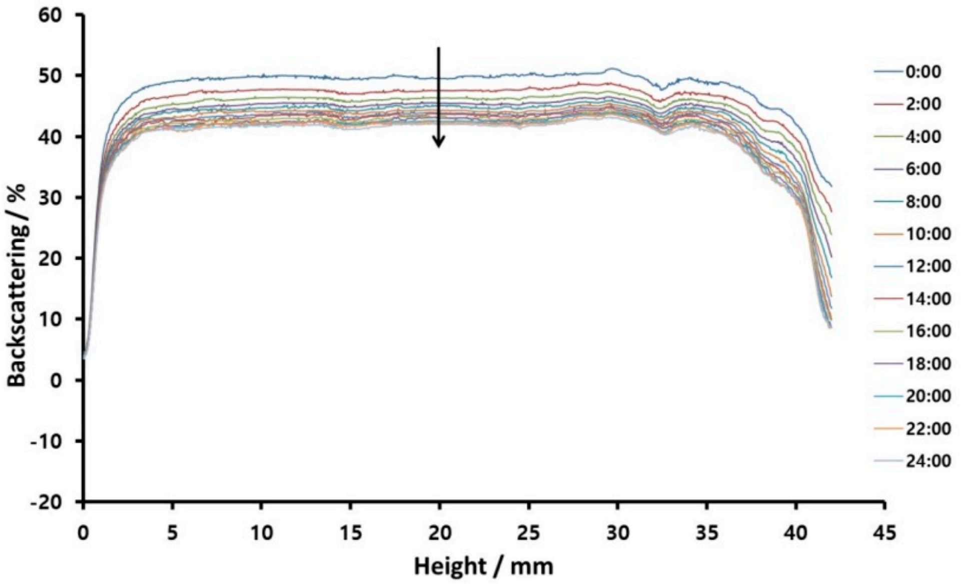

Figure S5. Backscattering change of the Pickering HIPE during 24 h after HIPE preparation (particle 5 wt%, PAAS 6 wt%, internal phase 80 vol%).

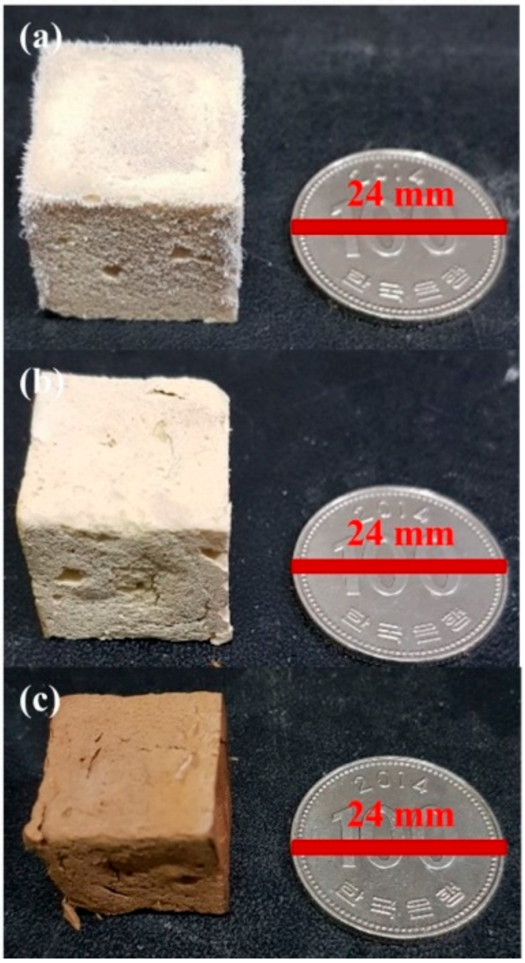

Figure S6. Photographs of products prepared by (a) freezing of HIPE, (b) lyophilization of HIPE and (c) thermal imidization after lyophilization.

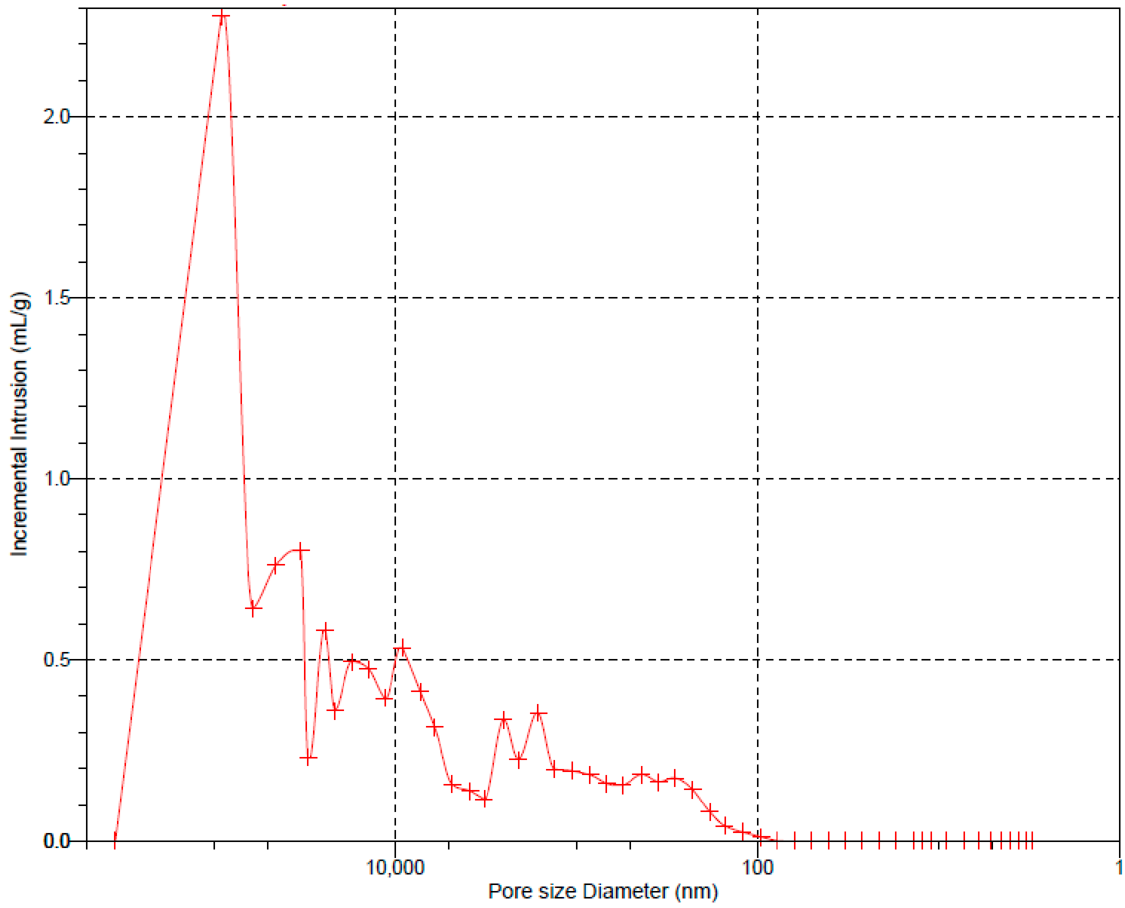

Figure S7. Pore size distribution of a polyHIPE sample prepared from a Pickering HIPE (5 wt% oligoimide particle concentration, 6 wt% PAAS concentration, and 80 vol% internal phase).
